# Image Processing Algorithms and Measures for the Analysis of Biomedical Imaging Systems Applications

**DOI:** 10.1155/2015/926921

**Published:** 2015-04-27

**Authors:** Karen Panetta, Sos Agaian, Jean-Charles Pinoli, Yicong Zhou

**Affiliations:** ^1^Tufts University, USA; ^2^University of Texas at San Antonio, USA; ^3^Ecole Nationale Supérieure des Mines, France; ^4^Computer and Information Science, University of Macau, Macau

The medical profession has changed dramatically in the past decade due to the advances in engineering innovations. Traditionally, medical doctors relied solely on their training and experience for diagnosis and interpretation of medical conditions captured through a variety of imaging techniques. Typically, these images are produced from CAT scan machines, MRI, or X-rays.

Today, computer aided techniques are designed to enhance the visual quality of biomedical images to better help humans discriminate regions of interest, such as the presence of cancer. Since these enhanced images are intended to assist the medical professional in making a diagnosis or to track the progress of a treatment, it is important that abnormalities presented in the images are actual objects and not artifacts resulting from the enhancement process that could lead to an inaccurate diagnosis.

Too much information presented can be distracting for the viewer and too little information presented could be disastrous for the patient, should a life-threatening abnormality be missed or filtered out. Therefore, the dilemma lies in answering the question: “What is the best image to use?”

The answer has always been subjective due to the perspective of the human observer and what constitutes the desired target object to be detected. For instance, some doctors looking at a scan may be interested in masses; another doctor may want to focus on calcifications or, even perhaps, the blood vessels.

In this special issue, articles are presented that address this question for several different biomedical image processing applications. Several novel quantifiable evaluation methods that can rank the quality of a processed image in accordance with human visual perception are introduced. These metrics are rigorously compared to evaluations obtained from human evaluators using the “Mean Opinion Score” (MOS), along with other standard benchmarking techniques.

The innovative approaches presented in this issue are sure to lead the biomedical image processing field beyond one of an “assistive technology” to one that creates a reliable, accurate autonomous technology for use in distance and robot vision applications. Achieving these goals will forge new groundbreaking paths that will reach into and serve remote populations of the world.

